# Data on affected cancer-related genes in pediatric t(12;21)-positive acute lymphoblastic leukemia patients harboring unbalanced der(6)t(X;6) translocations

**DOI:** 10.1016/j.dib.2016.06.060

**Published:** 2016-07-05

**Authors:** Eigil Kjeldsen

**Affiliations:** Hemodiagnostic Laboratory, Cancer Cytogenetics Section, Department of Hematology, Aarhus University Hospital

**Keywords:** Acute lymphatic leukemiat(12;21)-positive, Cancer genomics

## Abstract

The t(12;21)(p13;q22), leading to ETV6/RUNX1 fusion, is of importance for leukemogenesis in acute lymphoblastic leukemia but is not sufficient for the leukemic transformation. Acquired secondary chromosomal aberrations are necessary for overt leukemia but their complete nature and genes involved are still elusive. In our recent publication, “Oligo-based aCGH analysis reveals cryptic unbalanced der(6)t(X;6) in pediatric t(12;21)-positive acute lymphoblastic leukemia”, we identified acquired common concurrent regions with 6q deletion and Xq duplication E. Kjeldsen (2016) [1]. The present article provides data on genes that are associated with hematological malignancy and other cancers located in these common regions of chromosomal aberrations.

**Specifications Table**

**Table:** 

Subject area	*Biology*
More specific subject area	*Cancer genomics*
Type of data	*Tables*
How data was acquired	*Search was performed in the repository **Network of Cancer Genes***
Data format	*Analyzed*
Experimental factors	*Bone marrow samples of t(12;21)-positive ALLs were analyzed by oligo-based array-comparative genomic hybridization, which identified a group of patients with der(6)t(X;6).*
Experimental features	*A common region of duplication on Xq and a common region of deletion on 6q were determined in our recent publication and used for the repository search.*
Data source location	*Aarhus, Denmark*
Data accessibility	*Data is with this article*

**Value of the data**

Non-random concurrent Xq duplication and 6q deletion in pediatric t(12;21)-positive ALLs may point to important secondary aberrations for leukemogenesis.

16 out of 59 cancer-related genes located in the aberrant genomic regions were associated with hematological malignancy.

The findings add to the spectrum of acquired secondary aberrations in t(12;21)-positive ALLs.

## 1 Data

A search at the repository *Network of Cancer Genes* (NCG5.0) [2] revealed 27 cancer-related genes in the common region of duplication Xq25-qter (genomic pos. 121,657,255-155,232,907) of which seven genes are described to be associated with hematological malignancy, including *STAG2, SMARCA1, PHF6, MAGEC3, PASD1, DUSP9,* and *RPL10* (Table 1). A search for cancer-related genes in the common region of deletion on 6q16.1-qter (genomic pos. 95,958,513–171,025,515) revealed 32 genes of which nine genes are associated with hematological malignancy, including *PRDM1, FYN, ROS1, EYA4, SGK1, TNFAIP3, CCDC28A, ECT2L, and ESR1* (Table 2). The tables also summarize current knowledge of the function(s) of the proteins encoded by the involved genes.

## 2 Experimental design, materials and methods

By oligobased aCGH analysis of three pediatric t(12;21)-positive ALLs we recently identified a common region of duplication on Xq (genomic pos. 121,657,255–155,232,907) and a common region of deletion on 6q (genomic pos. 95,958,513–171,025,515) [1]. This genomic information was used to search the web-based *Network of Cancer Genes* (NCG5.0) [2]. The resulting information of genes located in these common regions of aberrations were listed in the tables without filtering.

## Figures and Tables

**Table 1:** Twenty-seven cancer related genes located in the common region of amplification in Xq25-qter, pos. 121,657,255–155,232,907.

**Genes**	**CytoBand**	**Cancer Type or site**^a^	**Protein function**	**Ref.**

***GRIA3***	Xq25	Pancreas	Cellular processes Signal transduction	[3]
***STAG2***	Xq25	Multiple Bladder **Leukemia** Ewing sarcoma Glioblastoma	Cell cycle	[4] [5] [6] [7] [8] [9], [10]
***DCAF12L2***	Xq25	Glioblastoma	No functional information	[11]
***SMARCA1***	Xq25	**Lymphoma**	Cell cycle Cellular metabolism DNA/RNA metabolism and transcription Development Regulation of intracellular processes and metabolism Regulation of transcription	[12]
***XPNPEP2***	Xq26.1	Lung	Cellular metabolism	[13]
***BCORL1***	Xq26.1	Glioma Intracranial	Cellular metabolism DNA/RNA metabolism and transcription Regulation of intracellular processes and metabolism Regulation of transcription	[14], [15]
***ELF4***	Xq26.1	Multiple	Cellular metabolism DNA/RNA metabolism and transcription Immune system response Regulation of intracellular processes and metabolism Regulation of transcription	[4]
***FRMD7***	Xq26.2	Glioblastoma	No functional information	[11]
***GPC3***	Xq26.2	Multiple	No functional information	[4]
***PHF6***	Xq26.2	Multiple **Leukemia**	Cellular metabolism DNA/RNA metabolism and transcription Regulation of intracellular processes and metabolism Regulation of transcription	[4] [7] [16]
***SAGE1***	Xq26.3	Pancreas	No functional information	[17]
***GPR112***	Xq26.3	Cholangio-carcinoma	Signal transduction	[18]
***RBMX***	Xq26.3	Endometrial	DNA/RNA metabolism and transcription Regulation of transcription	[19]
***ZIC3***	Xq26.3	Liver	Cellular metabolism DNA/RNA metabolism and transcription Development Regulation of intracellular processes and metabolism Regulation of transcription	[20]
***MAGEC3***	Xq27.2	**Leukemia** **Lymphoma**	No functional information	[16] [12]
***MAGEC1***	Xq27.1	Melanoma Head and neck	No functional information	[21] [22]
***MAGEC2***	Xq27.2	Head and neck	No functional information	[22]
***SPANXN2***	Xq27.3	Gastric	No functional information	[23]
***AFF2***	Xq28	Breast	Development	[19]
***PASD1***	Xq28	**Lymphoma**	Signal transduction	[24]
***MAGEA6***	Xq28	Pancreas	No functional information	[25]
***ATP2B3***	Xq28	Multiple	Cellular processes	[4]
***DUSP9***	Xq28	**Lymphoma**	Cellular metabolism Cellular processes Regulation of intracellular processes and metabolism Signal transduction	[12]
***RPL10***	Xq28	Multiple **Leukemia**	Cellular metabolism Regulation of transcription	[4] [16]
***PLXNA3***	Xq28	Neuroblastoma	Cell cycle Cell motility and interactions Development Regulation of intracellular processes and metabolism Signal transduction	[26]
***F8***	Xq28	Pancreas	Cell response to stimuli Immune system response Multicellular activities	[3]
***MTCP1***	Xq28	Multiple	No functional information	[4]

a Hematological malignancies are in **bold**.

**Table 2:** Thirty-two cancer related genes located in the common region of deletion on 6q16.1-qter, pos. 95,958,513–171,025,515.

**Genes**	**CytoBand**	**Cancer type or Site**^a^	**Protein function**	**Ref.**

***MCHR2***	6q16.2	Lung	Signal transduction	[27]
***PRDM1***	6q21	**Lymphoma**	Cellular metabolism DNA/RNA metabolism and transcription Regulation of intracellular processes and metabolism Regulation of transcription	[12]
***FOXO3***	6q21	Multiple	Cell Cycle Cellular metabolism DNA/RNA metabolism and transcription Development Multicellular activities Regulation of intracellular processes and metabolism Regulation of transcription	[4]
***FYN***	6q21	**Lymphoma**	Cell response to stimuli Cellular metabolism Cellular processes Immune system response Multicellular activities Signal transduction	[28]
***LAMA4***	6q21	Bladder Intracranial	Cell motility and interactions Development	[5] [14]
***DSE***	6q22.1	Liver	Cellular metabolism	[29]
***RFX6***	6q22.1	Glioblastoma	Cellular metabolism DNA/RNA metabolism and transcription Regulation of intracellular processes and metabolism Regulation of transcription	[11]
***ROS1***	6q22.1	Multiple Lung **Leukemia**	Cellular metabolism Cellular processes Signal transduction	[4], [27], [30]
***GOPC***	6q22.1	Multiple Lung	Cellular metabolism Cellular processes Regulation of intracellular processes and metabolism	[4] [31]
***TBC1D32***	6q22.31	Glioblastoma	Regulation of intracellular processes and metabolism Signal transduction	[32]
***PTPRK***	6q22.33	Melanoma	Cellular metabolism Cellular processes	[21]
***LAMA2***	6q22.33	Cholangio-carcinoma	Cell motility Development	[33]
***EYA4***	6q23.2	Colorectal Lung **Leukemia**	Cell response to stimuli Cellular metabolism Regulation of intracellular processes and metabolism Regulation of transcription	[34] [35] [36]
***SGK1***	6q23.2	**Lymphoma** Endometrial	Cell cycle Cellular metabolism Cellular processes	[24] [37] [38]
***MYB***	6q23.3	Multiple Glioma	Cellular metabolism DNA/RNA metabolism and transcription Regulation of intracellular processes and metabolism Regulation of transcription	[4] [39]
***BCLAF1***	6q23.3	Lung	Cell cycle Cellular metabolism DNA/RNA metabolism and transcription Development Regulation of intracellular processes and metabolism Regulation of transcription	[40]
***MAP7***	6q23.3	Colorectal	Cellular processes Development	[41]
***MAP3K5***	6q23.3	Melanoma	Cell cycle Cellular metabolism Cellular processes Development Regulation of intracellular processes and metabolism Signal tranduction	[42]
***TNFAIP3***	6q23.3	**Lymphoma**	Cell cycle Development Regulation of intracellular processes and metabolism Signal tranduction	[12] [43]
***CCDC28A***	6q24.1	**Leukemia**	No functional information	[44]
***ECT2L***	6q24.1	Multiple **Leukemia**	Signal transdcution	[4] [45]
***GRM1***	6q24.3	Lung Chondronmyxoid fibroma	Multicellular activities Signal transduction	[27] [46]
***ESR1***	6q25.1	Breast Endometrial **Leukemia**	Cell cycle Cellular metabolism DNA/RNA metabolism and transcription Development Regulation of intracellular processes and metabolism Regulation of transcription Signal transduction	[47] [48]
***SYNE1***	6q25.1-q25.2	Bladder Esophageal Nasopharyngeal Head and neck	Cellular processes Development	[5] [49] [50] [22]
***ARID1B***	6q25.3	Neuroblastoma Breast Lung Liver	Cellular metabolism DNA/RNA metabolism and transcription Regulation of intracellular processes and metabolism Regulation of transcription	[51] [52] [53] [54] [20]
***EZR***	6q25.3	Multiple	Cell motility and interactions Cellular processes Development Regulation of intracellular processes and metabolism	[4]
***IGF2R***	6q25.3	Pancreas Glioma	Cellular processes Signal transduction	[55] [56]
***MAP3K4***	6q26	Endometrial	Cellular metabolism Cellular processes Regulation of intracellular processes and metabolism Signal transduction	[57]
***QKI***	6q26	Glioma	Cellular metabolism Cellular processes DNA/RNA metabolism and transcription Regulation of intracellular processes and metabolism Regulation of transcription	[39]
***C6orf118***	6q27	Esophageal	No functional information	[49]
***FGFR1OP***	6q27	Multiple	Cell cycle Regulation of intracellular processes and metabolism	[4]
***MLLT4***	6q27	Multiple	Signal transduction	[4]

a Hematological malignancies are in **bold**.
