# Supplementary material for: Data on affected cancer-related genes in pediatric t(12;21)-positive acute lymphoblastic leukemia patients harboring unbalanced der(6)t(X;6) translocations
Source: Data Brief. 2016 Jul 5;8:894–903. doi: 10.1016/j.dib.2016.06.060 (PMC4961797; doi:10.1016/j.dib.2016.06.060)
Supplement: Supplementary file 1 — Supplementary material [file mmc1.doc]

# Potential conflicts of interests

The author has no conflicts of interest to declare.
